# Fgf10 Signaling in Lung Development, Homeostasis, Disease, and Repair After Injury

**DOI:** 10.3389/fgene.2018.00418

**Published:** 2018-09-25

**Authors:** Tingting Yuan, Thomas Volckaert, Diptiman Chanda, Victor J. Thannickal, Stijn P. De Langhe

**Affiliations:** Division of Pulmonary, Department of Medicine, Allergy and Critical Care Medicine, University of Alabama at Birmingham, Birmingham AL, United States

**Keywords:** Fgf10, regeneration, epithelium, fibrosis, injury

## Abstract

The lung is morphologically structured into a complex tree-like network with branched airways ending distally in a large number of alveoli for efficient oxygen exchange. At the cellular level, the adult lung consists of at least 40–60 different cell types which can be broadly classified into epithelial, endothelial, mesenchymal, and immune cells. Fibroblast growth factor 10 (Fgf10) located in the lung mesenchyme is essential to regulate epithelial proliferation and lineage commitment during embryonic development and post-natal life, and to drive epithelial regeneration after injury. The cells that express *Fgf10* in the mesenchyme are progenitors for mesenchymal cell lineages during embryonic development. During adult lung homeostasis, *Fgf10* is expressed in mesenchymal stromal niches, between cartilage rings in the upper conducting airways where basal cells normally reside, and in the lipofibroblasts adjacent to alveolar type 2 cells. Fgf10 protects and promotes lung epithelial regeneration after different types of lung injuries. An Fgf10-Hippo epithelial-mesenchymal crosstalk ensures maintenance of stemness and quiescence during homeostasis and basal stem cell (BSC) recruitment to further promote regeneration in response to injury. *Fgf10* signaling is dysregulated in different human lung diseases including bronchopulmonary dysplasia (BPD), idiopathic pulmonary fibrosis (IPF), and chronic obstructive pulmonary disease (COPD), suggesting that dysregulation of the FGF10 pathway is critical to the pathogenesis of several human lung diseases.

## Epithelial Fgf10 Signaling During Lung Development

Fibroblast growth factor 10 (Fgf10) was first detected using whole-mount *in situ* hybridization 20 years ago in the splanchnic mesoderm surrounding the foregut around E9.5 when the primary lung buds start to emerge. Lung primordial mesoderm-specific transcription factor Tbx4 defines the *Fgf10* expression domain, at both the anterior and posterior boundaries (Sakiyama et al., 2003). The importance of Fgf10 in lung development is well illustrated by the total failure of lung formation and perinatal lethality of *Fgf10* deficient mice (Min et al., 1998; Xu et al., 1998; Sekine et al., 1999). Even though Fgf10 binds with high affinity to Fgfr2b, it has a weaker affinity for Fgfr1b (Ohuchi et al., 2000). The *Fgf10* knockout phenotype is phenocopied in mice lacking *Fgfr2b* (Arman et al., 1999; De Moerlooze et al., 2000), which is highly expressed in respiratory epithelium from the early embryonic lung bud stages through late fetal lung development (Peters et al., 1992). Intriguingly, Fgfr2b has also been detected in the lung mesenchyme (Al Alam et al., 2015), but its mesenchymal function requires further investigation. Although Fgfr2b is a receptor for both Fgf7 and Fgf10 during lung development, *Fgf7* knockout mice do not exhibit an obvious lung defect (Guo et al., 1996), even though *Fgf7* is expressed in the developing lung mesenchyme starting at E14.5 (Mason et al., 1994). However, overexpression of *Fgf7* in mice using the human Sftpc promoter results in severe pulmonary malformations, including bronchial airway enlargement, cystic lung lesions and impaired branching morphogenesis leading to embryonic lethality (Simonet et al., 1995).

**FIGURE 1:**
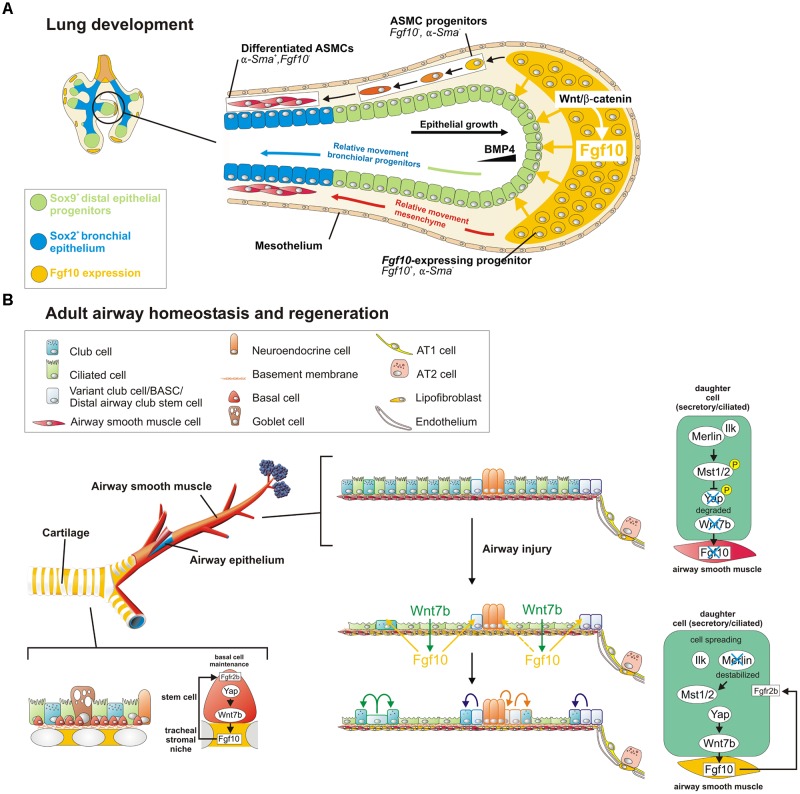
A Wnt7b-Fgf10 epithelial-mesenchymal crosstalk maintains distal epithelial progenitors during lung development and becomes reactivated in the adult lung to regenerate injured airway epithelium. **(A)** During the branching stage of lung development, Fgf10 is expressed by mesenchymal progenitor cells, which depends on Wnt/β-catenin signaling, and acts on the distal epithelium to induce Bmp4 and Sox9 expression to keep them in an undifferentiated state. As the epithelial tube grows toward the Fgf10 source, Sox9 + progenitors acquire more proximal positions, switch on Sox2 expression and acquire bronchial epithelial fate. Simultaneously, distal Fgf10-expressing airway smooth muscle (ASMC) progenitors encounter epithelial Bmp4 and Shh (not shown) causing them to stop expressing Fgf10 and differentiate into mature ASMCs as they relocate proximally. **(B)** In the adult, basal stem cells (BSCs) generate their own Fgf10-expressing niche mediated by Yap-Wnt7b, and their maintenance is critically dependent on Fgf10-Fgfr2b signaling. The non-cartilaginous airway epithelium is kept quiescent during homeostasis, by active Integrin-linked kinase (Ilk)-Hippo signaling, which prevents Fgf10 expression in ASMCs. In response to injury, surviving epithelial cells spread out, leading to a destabilization of Merlin and inactivation (dephosphorylation) of Hippo kinases Mst1/2. This increases nuclear Yap in spreading epithelial cells causing these cells to secrete Wnt7b. Epithelial-derived Wnt7b then acts on ASMCs to induce Fgf10 expression, which is required for epithelial regeneration. Solid cell borders represent lineage labels to follow the fate of epithelial cells in response to injury.

From E10.5 to E12.5, *Fgf10* expression is restricted to the distal lung mesenchyme at sites where branching occurs (Bellusci et al., 1997) and the ventral mesenchyme of the trachea (Sala et al., 2011; **Figure 1A**). For a long time, the localized pattern of *Fgf10* expression in the distal lung was thought to determine where new lung buds sprout. However, proper epithelial branching still occurs in developing *Fgf10^-/-^* lungs in which *Fgf10* is overexpressed in every cell. This indicates that the precise spatial organization of *Fgf10* expression is not required for the highly preserved and stereotypic branching morphogenesis. Hence, other mechanical and/or signaling pathways systems must be in place to control bud outgrowth. Instead, localized *Fgf10* expression in the distal mesenchyme is required to regulate epithelial lineage commitment (Volckaert et al., 2013) by maintaining the undifferentiated status of the distal Sox9-expressing epithelial progenitors and preventing them from differentiating into Sox2^pos^ bronchial epithelium (**Figure 1A**). *Fgf10* achieves this, in part, by activating epithelial β-catenin signaling through activation of Akt, which negatively regulates Sox2 expression (Volckaert et al., 2013). Indeed, Wnt/β-catenin signaling is important for the regulation of proximal-distal differentiation in the developing airway epithelium (De Langhe et al., 2005; Hashimoto et al., 2012; Ostrin et al., 2018). As the epithelium grows out, cells which become further and further displaced from the source of Fgf10 start to differentiate into Sox2^pos^ bronchial epithelium (Volckaert et al., 2013; Volckaert and De Langhe, 2014; **Figure 1A**). As a corollary, *Fgf10* hypomorphs and conditional *Fgf10* (*Dermo1-cre;Fgf10)* and *Fgfr2 (Sftpc-cre;Fgfr2)* mutants fail to maintain distal progenitors, resulting in a proximalized lung with impaired alveolar epithelial lineage formation and reduced capacity to produce surfactant proteins (Mailleux et al., 2005; Ramasamy et al., 2007; Abler et al., 2009). In addition, in lungs overexpressing *Fgf10* early on, distal epithelial progenitors fail to differentiate into bronchial epithelium (Volckaert et al., 2013). Taken together, these findings indicate that epithelial-mesenchymal interactions between Fgfr2b and its ligand Fgf10 is required for lung epithelial lineage commitment (Xu et al., 1998; Sekine et al., 1999; Ohuchi et al., 2000).

The localized expression of *Fgf10* in the trachea, on the other hand, drives submucosal gland (SMG) and basal cell development and their maintenance (Rawlins and Hogan, 2005; Volckaert et al., 2013; Volckaert et al., 2017). At the onset of lung and trachea initiation, Fgf10 is detected in the ventral mesenchyme of the trachea (Sala et al., 2011), and then becomes restricted to the intercartilage mesenchyme at later stages and into adulthood (Sala et al., 2011). Interestingly, although *Fgf10^-/-^* and *Fgfr2b^-/-^* embryos are born without lungs, they still develop a trachea (Sekine et al., 1999; De Moerlooze et al., 2000; Sala et al., 2011). SMGs are severely reduced in number and size in *Fgf10* heterozygotes (Jaskoll et al., 2005; Rawlins and Hogan, 2005). Abnormal function of SMGs of the upper respiratory tract are associated with severe/fatal asthma and cystic fibrosis later in life (Benayoun et al., 2003; Salinas et al., 2005). However, despite the significance of SMGs for human respiratory diseases, little is known about the mechanisms of Fgf10 signaling that controls their growth, differentiation, and homeostasis during early postnatal and adult life.

Overexpression of *Fgf10* at later stages of lung development, post-Sox2^pos^ bronchial epithelial specification, directs the differentiation of Sox2^pos^ proximal airway epithelium toward the p63/Krt5^pos^ basal cell lineage while blocking Foxj1^pos^ ciliated cell fate throughout the conducting airway (Volckaert et al., 2013). The cells that express *Fgf10* in the mesenchyme are themselves progenitors for airway and vascular smooth muscle cells as well as lipofibroblasts (LIFs) during embryonic development, and a subset of lung resident mesenchymal stem cells during adult life (Mailleux et al., 2005; Taniguchi et al., 2007; El Agha et al., 2014). Interestingly, Fgf10 also directly and indirectly orchestrates differentiation of these mesenchymal progenitors (El Agha and Bellusci, 2014; Chao et al., 2015). Epithelial BMP4, a target of Fgf10, controls the differentiation of cells arising from the distal mesenchymal *Fgf10*-expression domain into the airway smooth muscle cell (ASMC) lineage (Mailleux et al., 2005). In addition, *Fgf10* hypomorphs demonstrate defective formation of alveolar myofibroblasts (aMYFs) at different developmental stages (Mailleux et al., 2005; Ramasamy et al., 2007).

Starting at E16.5, Id2^pos^ Sox9^pos^ Sftpc^pos^ Pdpn^pos^ alveolar/bipotent epithelial progenitors give rise to alveolar type I and II (AT1/AT2) cells (Desai et al., 2014; Treutlein et al., 2014). Alveolar epithelial differentiation is coordinated by both mechanical forces and growth factors. In this context, it was recently shown that mechanical forces generated by fetal breathing movements stimulate AT1 cell differentiation, whereas Fgf10-mediated ERK1/2 signaling in distal progenitor cells prevents them from differentiating, thereby ensuring their AT2 fate (Li et al., 2018). In the mesenchyme, Gli^pos^ Pdgfra^pos^ mesenchymal progenitor cells give rise to aMYFs and LIFs (Li et al., 2015; Chao et al., 2016). Although aMYFs and LIFs are both derived from Gli1^pos^ Pdgfrα^pos^ mesenchymal progenitors, LIFs exhibit lower Pdgfrα^pos^ expression and higher levels of *Fgf10* expression in association with its receptors Fgfr1b and Fgfr2b. This suggests that different Fgfr and ligand profiles might mediate the direction of differentiation from Pdgfrα^pos^ mesenchymal progenitors toward LIF or aMYF (McGowan and McCoy, 2015). Interestingly, it has been shown that LIFs consist of both Fgf10^pos^ and Fgf10^neg^ subpopulations (Al Alam et al., 2015). *Fgf10* reduction in *Fgf10* hypomorphs as well as knockdown of *Fgfr2b* ligand *in vivo* led to significantly decreased expression of LIF marker Adrp at E18.5 in global LIF subpopulations (Fgf10^pos^ and Fgf10^neg^). This suggests that Fgf10 signals promote the formation of LIFs in an autocrine and/or paracrine fashion (Al Alam et al., 2015). Additionally, constitutive *Fgfr1b* knockouts and conditional partial loss of *Fgfr2b* in lung mesenchyme revealed that Fgfr1b and Fgfr2b are likely to play redundant roles in LIF formation (Al Alam et al., 2015). Finally, Apert syndrome mice, which exhibit a splicing switch defect resulting in increased mesenchymal Fgfr2b expression, demonstrate increased *Fgf10* expression and signaling in the mesenchyme. These mice display reduced epithelial branching, arrested development of terminal airways and an “emphysema like” phenotype in post-natal lungs resulting from decreased canonical Wnt signaling (De Langhe et al., 2006), likely due to sequestering of the Fgf10 ligand by the misexpressed Fgfr2b receptor.

## Fgf10 Signaling During Lung and Trachea Homeostasis

**FIGURE 2:**
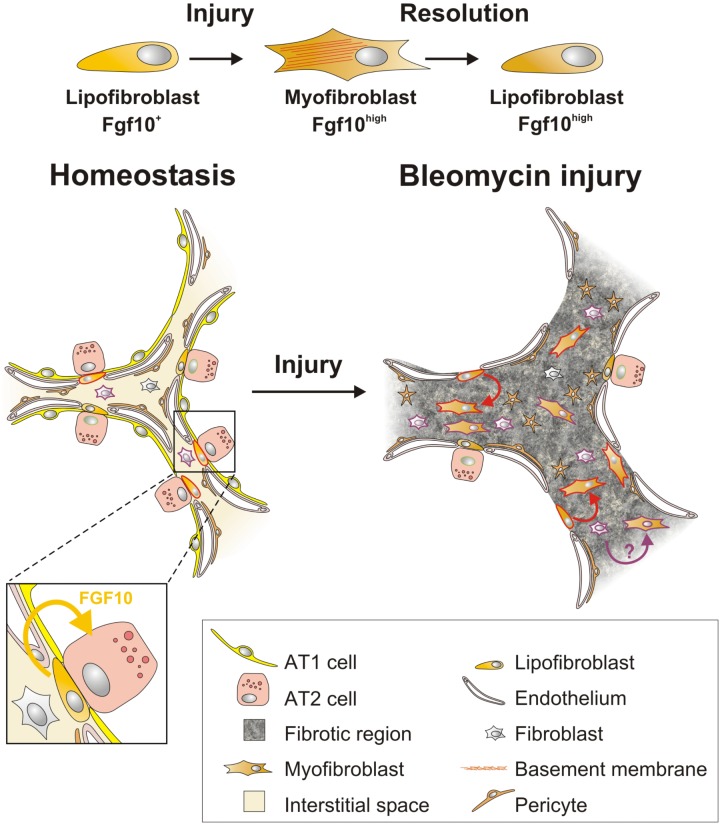
Fgf10-expressing lipofibroblasts are a source of activated myofibroblasts, which re-acquire lipogenic fate during fibrosis resolution. During homeostasis, lipofibroblasts (LIFs) express Fgf10 and are near alveolar type II (AT2) cells. In response to bleomycin-mediated alveolar epithelial injury, LIFs undergo a lipogenic to myogenic switch in fibroblastic phenotype and upregulate Fgf10 expression. Vice versa, during fibrosis resolution, myofibroblasts re-acquire lipofibroblast fate yet retain high Fgf10 expression. Solid cell borders represent lineage labels to follow the fate of (lipo)fibroblasts.

During homeostasis, adult mouse lungs harbor three main stem cell populations that maintain the lung epithelium: basal stem/progenitor cells (BSCs) in the cartilaginous airways, club cells in the conducting airways and subsets of AT2 cells in the alveoli (Rawlins et al., 2009; Rock et al., 2010; Barkauskas et al., 2013). During homeostasis *Fgf10* is expressed in mesenchymal stromal niches, between cartilage rings in the upper conducting airway where basal cells normally reside, and in the LIFs adjacent to AT2 cells in the alveoli (El Agha et al., 2014; **Figures 1B, 2**).

BSCs are progenitors for club, Tuft1/2, neuroendocrine and ionocyte cells (Rock et al., 2009, 2010; Montoro et al., 2018). In the developing trachea, Fgf10 secreted by the inter-cartilage stromal tissue is involved in the development and maintenance of BSCs (**Figure 1B**). Overexpression of *Fgf10* in the trachea leads to BSC amplification whereas overexpressing *Fgf10* in adult club cells extends the BSC niche and induces club and BSC hyperplasia in conducting airways (Volckaert et al., 2017). Consistently, both Fgfr2b ligands Fgf7 and Fgf10 can promote basal cell colony expansion *in vitro* (Balasooriya et al., 2017). Furthermore, Fgfr2b signaling in the trachea is required for BSC maintenance during adult lung homeostasis (Volckaert et al., 2013, 2017). Even loss of one copy of *Fgfr2* in adult mouse airway BSCs is sufficient to reduce BSC self-renewal with cells quickly becoming senescent (Balasooriya et al., 2017). Interestingly, conditional deletion of *Fgfr1* or *Spry2* specifically in adult mouse tracheal BSCs using the *Krt5* promoter causes increased ERK/AKT signaling and BSC proliferation and a block in ciliated cell differentiation (Balasooriya et al., 2016), possibly due to increased Fgfr2b signaling caused by a lack of Spry2 activation by Fgfr1. This phenotype resembles that of tracheas overexpressing *Fgf10*, suggesting that this Fgfr1-SPRY2 signaling axis might function to antagonize Fgf10/Fgfr2b/ERK/AKT signaling, which is required for maintaining quiescence and restricting BSC proliferation in the steady-state airway epithelium *in vivo*.

## Fgf10 Signaling in Repair of the Injured Lung

Recent studies indicate that Fgf10 prevents lung injury and promotes lung epithelial regeneration after various stresses, including bleomycin-induced alveolar epithelial lung injury (Gupte et al., 2009), influenza-induced acute respiratory distress syndrome (Quantius et al., 2016), high altitude pulmonary edema (She et al., 2012), LPS-induced lung injury (Tong et al., 2014), mechanical ventilation induced lung injury (Bi et al., 2014), ischemia-reperfusion lung injury (Fang et al., 2014), hyperoxia-induced neonatal lung injury (Chao et al., 2017), and naphthalene injury (Volckaert et al., 2011). In a post-pneumonectomy model, Fgfr2b ligands were shown to be required for aMYF formation during the regenerative response (Chen et al., 2012).

In the bleomycin model of pulmonary fibrosis, *Fgf10* overexpression in the alveolar epithelium of *Sftpc-rtTA;Tet-Fgf10* mice attenuates fibrosis through inhibition of TGF-β and improved survival of AT2 cells This indicates that Fgf10 has a protective as well as regenerative effect on epithelial progenitor cells (Gupte et al., 2009). Similarly, Fgf10 via the Grb2-SOS/Ras/Raf-1/MAPK pathway attenuates H_2_O_2_-induced alveolar epithelial DNA damage (Upadhyay et al., 2004). Overexpression of a dominant-negative Fgfr2 receptor (dnFgfr), specifically in the lung epithelium, inhibited retinoic acid-induced alveolar regeneration in association with increased PDGFRα^pos^ and reduced expression of SMA in interstitial myofibroblasts (Perl and Gale, 2009). Intra-tracheal administration of Fgf10 attenuates lipopolysaccharide (LPS)-induced acute lung injury with increased AT2 proliferation (Tong et al., 2014). Lung resident mesenchymal stromal cells (MSCs) isolated from Fgf10 pretreated rats are protected against LPS-induced acute lung injury (Tong et al., 2016). However, the mechanism underlying these protective effects of Fgf10 signaling during injury and regeneration in adult lung have not yet been fully elucidated.

*Fgf10*-expressing cells were identified as a subset of LIF progenitors during embryonic development (El Agha et al., 2014). *Fgf10*-expressing LIFs have been shown to differentiate into activated MYFs upon bleomycin injury, while simultaneously upregulating their *Fgf10* expression levels (El Agha et al., 2017). *Fgf10*-expressing MYFs dedifferentiate back into LIFs but do not downregulate their *Fgf10* expression levels during the resolution phase of lung fibrosis (El Agha et al., 2017) suggesting that they retain a memory of the injury which might protect against further injury. This supports the concept that LIFs serve as a source of activated MYFs during fibrogenesis which revert back to LIFs during fibrosis resolution (El Agha et al., 2017; **Figure 2**).

Naphthalene injury is a well-established injury model to study conducting airway epithelial regeneration by selectively ablating club cells except for a few naphthalene-resistant club stem cells located at bronchoalveolar duct junctions (BADJs) and adjacent to neuroendocrine bodies (NEBs). In the adult lung, *Fgf10* is not expressed in mature ASMCs during homeostasis (**Figure 1B**). However, upon conducting airway epithelial injury, when surviving differentiated epithelial cells spread in an attempt to maintain barrier function, they downregulate their Hippo pathway to drive Yap into the nucleus, and induce the secretion of Wnt7b. Epithelial-derived Wnt7b, in turn, induces Lgr6^pos^ ASMCs to release Fgf10 (Volckaert et al., 2011, 2017; Volckaert and De Langhe, 2014; Lee et al., 2017), which activates Notch and β-catenin signaling in surviving club cells to drive their amplification to promote regeneration (Volckaert et al., 2011; Lee et al., 2017; **Figure 1B**). Together, these findings provide strong evidence that ASMCs function as a niche for conducting airway epithelial stem cells. Besides club cell regeneration, the induction of *Fgf10* expression by the ASMC niche in non-cartilaginous airways extends the BSC niche, allowing the recruitment of tracheal BSCs and/or driving the differentiation of Sox2^pos^p63^pos^Krt5^neg^ progenitors along the BSC lineage (Volckaert et al., 2017; Yang et al., 2018). In summary, the Fgf10-Hippo epithelial-mesenchymal crosstalk ensures maintenance of stemness and quiescence during homeostasis and recruitment of BSCs to promote regeneration in response to injury (Volckaert et al., 2017; **Figure 1B**).

A similar tonic Hedgehog signal maintains lung airway epithelial and mesenchymal quiescence in the distal mouse airways (Peng et al., 2015). In this model, loss of Hedgehog signaling drives regeneration in response to naphthalene-induced epithelial injury via a mesenchymal feedback mechanism, and deregulation of hedgehog during naphthalene induced epithelial lung injury leads to aberrant repair and regeneration (Peng et al., 2015). These findings imply that the Wnt-Fgf10 epithelial-mesenchymal cross-talk and Shh pathway may function as an interactive signaling network in airway and alveolar remodeling responses to chronic injury in asthma, chronic obstructive pulmonary disease (COPD) and pulmonary fibrosis.

## Fgf10 Signaling in Human Lung Diseases

Several syndromic craniosynostoses have been associated with dominantly acting mutations of *FGFR1*, *FGFR2*, and *FGFR3* (Hajihosseini et al., 2001). *FGFR2B* is up-regulated in cultured fibroblasts of some Apert’s and Pfeiffer’s syndrome patients (Oldridge et al., 1999). Gain-of-Fgfr2b function mice *Fgfr2c^+/Δ^* show phenotypic resemblance to Apert’s and Pfeiffer’s syndromes, including visceral and growth defects, neonatal growth retardation and death, coronal synostosis, ocular proptosis, precocious sternal fusion, and abnormalities in secondary branching in lung and kidney that undergo branching morphogenesis (Hajihosseini et al., 2001; De Langhe et al., 2006).

In humans, haploinsuffiencies for *FGF10* or *FGFR2B* result in autosomal dominant aplasia of lacrimal and salivary glands and lacrimo auriculo-dentodigital syndrome, respectively (Entesarian et al., 2005; Klar et al., 2011). In the former syndrome, patients exhibit irreversible airway obstruction, indicating that genetic variants affecting the FGF10 signaling pathway are important determinants of lung function which ultimately contribute to COPD (Klar et al., 2011). Notably, an airway branch variant with absence of the right medial-basal airway associated with polymorphisms within the *FGF10* gene is associated with COPD among smokers (Smith et al., 2018). Interestingly, increased nuclear YAP levels, along with FGFR2B and WNT7b expression, were observed in squamous metaplastic areas within the airway epithelium of COPD subjects (Volckaert et al., 2017), suggesting that the Hippo pathway is inactivated to induce FGF10 expression and BSC amplification in human COPD.

Bronchopulmonary dysplasia (BPD) is a chronic pulmonary disease of prematurely born infants characterized by arrested alveolar development (Chao et al., 2017). BPD biopsy samples show reduced *FGF10* expression (Benjamin et al., 2007), implicating that FGF10 signaling may be involved in BPD. By using hyperoxia-induced neonatal lung injury from post-natal day 0 (P0) to P8 as a mouse model of BPD, Chao et al. (2017) have shown that *Fgf10* deficiency causes lethality from P5 in *Fgf10^+/^*^-^ pups due to impaired AT2 formation after hyperoxic injury. In this study, overexpression of a secreted dominant negative *Fgfr2b*, demonstrated that post-natal deficiency of Fgfr2b ligands in the context of hyperoxia-exposure causes decreased *Sftpc* expression and eventually leads to significant lethality. This indicates that Fgfr2b ligands are important for repair after hyperoxia exposure in neonatal lung.

Idiopathic pulmonary fibrosis (IPF) is a chronic interstitial lung disease characterized by the loss of alveolar epithelial integrity, progressive invasion of the lung parenchyma by myofibroblasts and increased extracellular matrix (ECM) deposition leading to respiratory failure, and death often within 5 years of diagnosis (Thannickal et al., 2004; King et al., 2011; Steele and Schwartz, 2013; Yang et al., 2013). Gene expression profiles of MSCs from IPF patient lungs revealed that *FGF10* expression in MSCs is suppressed in IPF subjects with progressive disease, along with upregulation of both TGF-β1 and SHH signaling. This suggests that *FGF10* deficiency is a potentially critical factor in disease progression (Chanda et al., 2016). However, recently it has been shown that FGF10 is significantly upregulated at both mRNA and protein level in IPF lungs compared to the donor lungs, especially in dense fibrotic islands where ACTA2^pos^ cells accumulate (El Agha et al., 2017).

## Conclusion

Fgf10 signaling is essential for lung development and adult stem cell maintenance. Important questions remain regarding the mechanisms that regulate *Fgf10* expression in the niche to unleash the full therapeutic potential of Fgf10. In addition, very little is known about the importance of FGF10 signaling in human lung development and homeostasis. During homeostasis, BSCs are restricted to the cartilaginous airway in mice as they require Fgfr2b signaling for their maintenance, whereas in humans they can be found deep in the lung. However, upon different types of injury BSCs are deployed throughout the mouse lung as ASMCs in the non-cartilaginous airways re-express *Fgf10* to regenerate the airway epithelium. It is therefore likely that the apparent restricted BSC pattern in the mouse lung is due to it being housed in a fairly sterile environment rather than constantly being exposed to environmental insults as is the case for humans.

## Author Contributions

TY, TV, and SDL wrote the manuscript. DC and VT edited the manuscript.

## Conflict of Interest Statement

The authors declare that the research was conducted in the absence of any commercial or financial relationships that could be construed as a potential conflict of interest.
